# Genetic characterization of third- or fourth-generation cephalosporin-resistant avian pathogenic *Escherichia coli* isolated from broilers

**DOI:** 10.3389/fvets.2022.1055320

**Published:** 2022-11-25

**Authors:** Hyo Jung Kang, Suk-Kyung Lim, Young Ju Lee

**Affiliations:** ^1^College of Veterinary Medicine and Zoonoses Research Institute, Kyungpook National University, Daegu, South Korea; ^2^Bacterial Disease Division, Animal and Plant Quarantine Agency, Gimcheon, South Korea

**Keywords:** avian pathogenic *E. coli*, third-or fourth-generation cephalosporin resistance, genetic characteristics, CRISPR typing method, multidrug resistance (MDR)

## Abstract

The third- or fourth-generation cephalosporins (3GC or 4 GC) are classified as “critically important antimicrobials for human medicine” by WHO, but resistance to these drugs is increasing rapidly in avian pathogenic *E. coli* (APEC). This study investigated the distribution and genetic characteristics of 3GC- or 4 GC-resistant APEC isolates from five major integrated broiler operations in Korea. The prevalence of 3GC- or 4GC-resistant APEC isolates in 1-week-old broilers was the highest in farms of operation C (53.3%); however, the highest prevalence of these isolates in 4-week-old broilers was the highest on the farms of operation A (60.0%), followed by operations E (50.0%) and C (35.7%). All 49 3GC- or 4GC-resistant APEC isolates had at least one β-lactamase-encoding gene. The most common β-lactamase-encoding genes was extended-spectrum β-lactamase gene, *bla*_CTX−M−15_, detected in 24 isolates (49.0%), followed by *bla*_TEM−1_ (32.7%). Sixteen isolates (32.7%) harbored class 1 integrons, and four isolates (8.2%) showed different gene cassette-arrangements. However, only 1 of 26 isolates harboring class 2 integrons carried a gene cassette. Furthermore, both CRISPR 1 and 2 arrays were detected in most isolates (36 isolates; 73.5%), followed by CRISPR 2 (18.4%) and CRISPR 1 (4.1%). Interestingly, CRISPR 2 was significantly more prevalent in multidrug resistant (MDR)-APEC isolates than in non-MDR APEC isolates, whereas CRISPR 3 and 4 were significantly more prevalent in non-MDR APEC isolates (each 11.1%; *p* < 0.05). None of the protospacers of CRISPR arrays were directly associated with antimicrobial resistance. Our findings indicate that the distribution and characteristics of 3GC or 4GC-resistant APEC isolates differed among the integrated broiler operations; moreover, improved management protocols are needed to control the horizontal transmission of 3GC or 4GC-resistant APEC isolates.

## Introduction

*Escherichia coli* (*E. coli*) is one of the most common facultative gram-negative bacteria found in the gastrointestinal tract of humans and animals (1), but certain strains of *E. coli* are responsible for a wide range of infections (2). In particular, avian colibacillosis is an important infectious disease caused by avian pathogenic *E. coli* (APEC) (3). APEC is defined as an extra-intestinal pathogenic *E. coli* (ExPEC) that cause diverse local and systemic infections in poultry, including chicken, turkeys, ducks, and many other avian species (4). In many countries, it results in large economic losses, which are attributable to early mortality and reduced productivity caused by a complex of severe respiratory and systemic diseases in chickens (5). Moreover, previous studies indicate that APEC can be a source of genes for other ExPEC strains that impact human medicine, such as uropathogenic *E. coli* and newborn meningitis-causing *E. coli* (6). There are certain APEC subgroups that has to be considered as potential zoonotic agents, implying the importance of APEC as a source of future zoonosis (7).

In modern broiler production, diverse antimicrobials, including β-lactams, aminoglycosides, and fluoroquinolones, are the predominant alternatives for treating colibacillosis (3). However, the use of such antimicrobials has contributed to the emergence of antimicrobial resistance globally (8). In particular, the rates of resistance to third- or fourth-generation cephalosporins (3GCs or 4GCs, respectively) which are classified as “critically important antimicrobials for human medicine” by WHO (9), in *E. coli* are increasing rapidly (10, 11) because of the increased prevalence of plasmid-encoded enzymes, such as extended-spectrum β-lactamases (ESBLs) and plasmid-mediated AmpC (pAmpC) β-lactamases (12). *E. coli* isolates that produce ESBLs and/or pAmpC β-lactamases are reported to be resistant to extended-spectrum cephalosporins such as 3GCs and 4GCs, crucial antimicrobials for human and animal health (13).

The broiler chicken industry operates through several large integrated operations. In Korea, 94.8% of the broilers are produced by integrated operations, and the top five integrated operations account for 51.4% of the market share (14). Thus, intensively reared broilers are exposed to routine prophylactic and therapeutic antimicrobial treatments; moreover, previous studies have reported that antimicrobial resistance patterns in APEC vary according to the integrated broiler operations (15, 16). Therefore, this study investigated the distribution, characteristics, and results of the CRISPR array typing, a novel typing method, of 3GC- or 4GC-resistant APEC isolates from five major integrated broiler operations in Korea.

## Materials and methods

### Sampling and bacterial identification

Chickens that died at 1 or 4 weeks of age at 74 broiler farms of five integrated operations were transferred to the laboratory for the isolation of APEC. Isolation and identification of *E. coli* were performed following the standard microbiological protocols published by the Ministry of Food and Drug Safety (17). Briefly, liver and spleen swab samples were collected in 10 ml of tryptic soy broth (TSB; BD Biosciences, San Jose, CA, USA), and incubated at 37°C for 18–24 h. The enriched broth was streaked onto MacConkey agar (BD Biosciences) and incubated at 37°C for 18–24 h. Suspected colonies were confirmed using polymerase chain reaction (PCR) with specific primers as described previously (18). If APEC isolates from the same farm displayed the same antimicrobial susceptibility patterns, one isolate was selected randomly.

### Antimicrobial susceptibility testing

Based on the Clinical and Laboratory Standards Institute (CLSI) guidelines (CLSI, 2021) (19), all isolates were investigated for antimicrobial resistance *via* the disk diffusion test with the following discs (BD Biosciences): ampicillin (AM, 10 μg), amoxicillin-clavulanate (20 μg), chloramphenicol (30 μg), ceftazidime (CAZ, 30 μg), cephalothin (CF, 30 μg), ciprofloxacin (5 μg), cefotaxime (CTX, 30 μg) cefuroxime (CXM, 30 μg), cefazoline (CZ, 30 μg), cefepime (FEP, 30 μg), cefoxitin (FOX, 30 μg), gentamicin (10 μg), imipenem (10 μg), nalidixic acid (30 μg), trimethoprim/sulfamethoxazole (1.25 μg), and tetracycline (30 μg). The minimum inhibitory concentrations (MIC**s**) of CAZ, CF, CTX, CXM, CZ, ceftiofur (EFT), FEP and FOX at concentrations of 0.5–512 μg/mL were determined *via* standard agar dilution methods using Mueller-Hinton agar (BD Biosciences) according to the CLSI recommendations (2021) (19). Multidrug-resistance (MDR) was defined as acquired resistance to at least one agent from three or more antimicrobial classes (20).

### Detection of β-lactamase-encoding genes

PCR amplification was performed to detect β-lactamase-encoding genes in 49 APEC isolates that exhibited resistance to 3GCs or 4 GCs. Primers for *bla*_CTX−M_, *bla*_TEM_, *bla*_SHV_, *bla*_OXA_, and pAmpC genes were used as described previously (21–23). The PCR products were purified using a QIAquick PCR purification kit (Qiagen, Hilden, Germany) and sequenced using an automatic sequencer (Cosmogenetech, Seoul, Korea). The sequences were then compared with those in the GenBank nucleotide database using the Basic Local Alignment Search Tool (BLAST) program available at the National Center for Biotechnology Information website (www.ncbi.nlm.nih.gov/BLAST).

### Detection of integrons and gene cassettes

The presence of the integrase genes *intl1* and *intl2* in 49 3GC- or 4GC-resistant APEC isolates was also detected using PCR as described previously (24, 25). APEC isolates harboring the integrase genes were tested for gene cassettes (25). The PCR products of the gene cassettes were sequenced as described previously, and the obtained sequences were compared with those in the GenBank using the BLAST program available at the National Center for Biotechnology Information website (www.ncbi.nlm.nih.gov).

### CRISPR locus sequence typing and spacer analysis

Four CRISPR loci in 49 3GC- or 4GC-resistant APEC isolates were screened using PCR as described previously (26). The PCR products were purified and sequenced according to the abovementioned procedure. The sequences were analyzed using CRISPRFinder (https://crispr.i2bc.paris-saclay.fr/Server/), as described by Grissa et al. (27), and only spacers were obtained for this study. The names and full sequences of all the spacers from this study are listed in Supplementary Table S1. The CRISPRTarget program (http://crispr.otago.ac.nz/CRISPRTarget/crispr_analysis.html) with a cut off value of 29 and nucleotide BLAST (https://blast.ncbi.nlm.nih.gov/Blast.cgi) were used to detect protospacers derived from phages or plasmids (28).

### Statistical analysis

The Statistical Package for Social Science version 25 (IBM SPSS Statistics for Windows, Armonk, NY, USA) was used for statistical analysis, which included the independent samples *t*-test, Fisher's exact test, and Pearson's chi-square test with Bonferroni correction as described previously (10, 29). Differences were considered significant at *p* < 0.05.

## Results

### Prevalence of 3GC- or 4GC-resistant APEC isolates

The number of chicken farms confirmed with colibacillosis and the distribution of farms with 3GC- or 4GC-resistant APEC isolates among five integrated operations are presented in Table 1. The highest prevalence of farms confirmed with colibacillosis in 1-week-old broilers 100% and the lowest was 85.7%, without significant differences among operations. However, the highest prevalence in 4-week-old broilers was detected at operation C (93.3%), %), followed by operations D (85.7%), E (80.0%), B (75.0%), and A (33.3%), with significant differences among the operations (*p* < 0.05). Regarding the prevalence of 3GC- or 4GC-resistant APEC isolates, 1-week-old broilers showed the highest resistance on the farms of operation C (53.3%), but the prevalence in 4-week-old broilers was the highest on the farms of operation A (60.0%), followed by operations E (50.0%) and C (35.7%).

**Table 1:** Distribution of third- or fourth- generation cephalosporin-resistant avian pathogenic *E. coli* isolates from five integrated broiler operations.

**Integrated broiler operation**	**No. (%) of farms confirmed with colibacillosis**		**No. (%) of farms with 3rd or 4th-generation cephalosporin resistant isolates** ^**+**^	
	**1 week**	**4 weeks**	**1 week**	**4 weeks**
A (*n =* 15)^†^	13 (86.7)	5 (33.3)^B^	3 (23.1)	3 (60.0)
B (*n =* 20)	19 (95.0)	15 (75.0)^A, B^	3 (15.8)	4 (26.7)
C (*n =* 15)	15 (100.0)	14 (93.3)^A^	8 (53.3)	5 (35.7)
D (*n =* 14)	12 (85.7)	12 (85.7)^A^	2 (16.7)	1 (8.3)
E (*n =* 10)	10 (100.0)	8 (80.0)^A, B^	3 (30.0)	4 (50.0)
Total (*n =* 74)	69 (93.2)	54 (73.0)	19 (27.5)	17 (31.5)

Values with different superscript letters represent significant differences among five integrated broiler operations, and significant difference of farms with 3rd and 4th-generation cephalosporin resistant isolates was absent between one week and four weeks old broilers (*p* < 0.05).

† No. of farms included in this study.

### Characteristics of 3GC- or 4GC-resistant APEC isolates

The genotypic and phenotypic characteristics of the 49 3GC- or 4GC-resistant APEC isolates are presented in Table 2. All 49 APEC isolates carried at least one β-lactamase-encoding gene. The most common β-lactamase-encoding gene was the ESBL gene, *bla*_CTX−M−15_, which was detected in 24 isolates (49.0%), followed by *bla*_TEM−1_ (32.7%), *bla*_CTX−M−1_ (14.3%), *bla*_CTX−M−14_ (14.3%), *bla*_SHV−12_ (6.1%), *bla*_CTX−M−27_ (4.1%), *bla*_OXA−1_ (4.1%), and *bla*_TEM−135_ (2.0%). Additionally, the pAmpC β-lactamase gene *bla*_CMY−2_, was detected in two isolates (4.1%).

**Table 2:** Characteristics of the 49 third- or fourth- generation cephalosporin-resistant avian pathogenic *E. coli* from five integrated broiler operations.

**Integrated broiler operation**	**Age (week)**	**Strain**	**Non-βlactam resistance**	**β-lactamase gene(s)**	**Minimum inhibitory concentrations (**μ**g/mL)**								***Int*I gene**	**Cassette array**	***IntII* gene**	**Cassette array**	**CRISPR array**
					**CZ**	**CF**	**FOX**	**CXM**	**CTX**	**CAZ**	**EFT**	**FEP**					
A	1	W2-L-2	NA, CIP	*bla*_CTX−M−1_	≥512	≥512	8	≥512	256	1	≥512	2	–	–	+	–	1, 2
A	1	W2-L-6	NA, CIP	*bla*_CTX−M−15_	≥512	≥512	16	≥512	≥512	64	≥512	256	–	–	+	–	1, 2
A	1	W5-L-10	TE, NA, CIP	*bla*_CTX−M−15_	≥512	≥512	8	≥512	≥512	32	≥512	256	–	–	+	–	1, 2
A	1	W12-L-6	NA, CIP, CHL	*bla*_CTX−M−15_ + *bla*_TEM1_	≥512	≥512	16	≥512	≥512	64	≥512	256	–	–	+	–	1, 2
A	4	AW-3-L-3	TE, SXT, NA, IPM, CIP, GM, CHL	*bla*_CTX−M−15_	≥512	≥512	16	≥512	≥512	16	≥512	128	–	–	+	–	2
A	4	AW-5-L-1	NA, CIP	*bla*_CTX−M−15_	≥512	≥512	16	≥512	≥512	32	≥512	256	–	–	–	–	1, 2
A	4	AW-5-L-3	NA, CIP, CHL	*bla*_CTX−M−15_ + *bla*_TEM1_	≥512	≥512	16	≥512	≥512	≥512	≥512	256	–	–	–	–	1, 2
A	4	AW-11-L-2	NA	*bla*_CTX−M−15_ + *bla*_TEM1_	≥512	≥512	4	≥512	≥512	32	≥512	256	+	–	–	–	1, 2
A	4	AW-11-L-2	TE, SXT, NA, CIP	*bla*_CTXM−15_ + *bla*_TEM1_	≥512	≥512	4	≥512	≥512	32	≥512	256	+	–	–	–	1, 2
B	1	D8-L-5	TE, NA, CIP, CHL	*bla*_SHV−12_	256	≥512	16	256	256	≥512	128	1	–	–	–	–	1, 2
B	1	D9-L-10	TE, NA, CIP, CHL	*bla*_SHV−12_	256	≥512	8	≥512	256	256	128	1	–	–	+	–	1, 2
B	1	D15-L-3	NA, CIP, GM, CHL	*bla*_CTX−M−14_ + *bla*_TEM1_	≥512	≥512	16	≥512	≥512	8	≥512	64	–	–	–	–	1
B	4	AD-2-L-1	SXT, NA, CIP, GM	*bla*_CTX−M−15_	≥512	≥512	8	≥512	≥512	32	≥512	128	+	–	+	–	1, 2
B	4	AD-7-L-4	TE, NA, CIP, CHL	*bla*_TEM1_ + *bla*_CMY−2_	≥512	≥512	128	≥512	≥512	32	≥512	256	–	–	+	*estX* + *sat2* + *sat1*	1, 2
B	4	AD-16-L-1	TE, SXT, NA, CIP, CHL	*bla*_CTX−M−15_ + *bla*_CMY−2_	≥512	≥512	32	≥512	≥512	64	≥512	256	+	–	+	–	2
B	4	AD-17-L-2	TE, NA, CIP, CHL	*bla*_CTX−M−15_	≥512	≥512	4	≥512	≥512	4	256	16	–	–	+	–	2
C	1	H1-L-5	NA, CIP, CHL	*bla*_CTX−M−15_ + *bla*_TEM1_	≥512	≥512	8	≥512	≥512	16	≥512	128	–	–	+	–	1, 2
C	1	H1-L-7	CIP	*bla*_TEM1_	≥512	≥512	8	≥512	≥512	4	128	4	+	–	–	–	2
C	1	H1-L-9	CIP	*bla*_TEM1_	≥512	≥512	8	≥512	128	4	64	256	+	–	+	–	–
C	1	H4-L-3	TE, SXT, NA, CIP	*bla*_CTX−M−14_	≥512	≥512	8	≥512	≥512	8	≥512	256	+	–	+	–	1, 2
C	1	H4-L-4	TE, NA, CIP	*bla*_CTX−M−1_	≥512	≥512	8	≥512	≥512	4	≥512	256	–	–	+	–	1, 2
C	1	H5-L-5	NA, CIP	*bla*_CTX−M−1_	≥512	≥512	8	≥512	≥512	2	≥512	128	–	–	+	–	1, 2, 3, 4
C	1	H6-L-3	SXT, NA, CIP, GM, CHL	*bla*_CTX−M−14_	≥512	64	8	≥512	≥512	1	≥512	128	+	–	–	–	1, 2
C	1	H6-L-4	TE, SXT, NA, GM, CHL	*bla*_TEM1_	16	≥512	8	128	128	1	0.5	0.5	+	*dfr*A12 + *aad*A1+*aad*A2	+	–	1, 2
C	1	H7-L-1	SXT, NA, CIP	*bla*_CTX−M−15_	≥512	≥512	8	≥512	≥512	8	≥512	256	+	*dfrA12*+*aadA1*+*aadA2*+*aadA22*	–	–	1, 2
C	1	H7-L-2	SXT, NA, CIP	*bla*_CTX−M−15_	≥512	≥512	8	≥512	≥512	16	≥512	256	+	*dfrA12*+*aadA1*+*aadA2*+*aadA22*	–	–	2
C	1	H8-L-2	TE, SXT, NA	*bla*_CTX−M−15_	≥512	≥512	8	≥512	≥512	32	≥512	256	+	–	–	–	2
C	1	H8-L-7	TE, NA	*bla*_CTX−M−15_	≥512	≥512	8	≥512	≥512	8	≥512	256	–	–	–	–	2
C	1	H9-L-2	TE, NA, CHL	*bla*_CTX−M−15_ + *bla*_TEM135_	≥512	≥512	8	≥512	≥512	16	≥512	256	–	–	+	–	1, 2
C	1	H9-L-7	SXT, NA, CIP, GM, CHL	*bla*_TEM1_	≥512	≥512	128	≥512	256	2	64	4	–	–	–	–	2
C	1	H12-L-6	NA, CIP, CHL	*bla*_CTX−M−1_	≥512	≥512	8	≥512	≥512	4	≥512	128	–	–	+	–	1, 2
C	4	AH-3-L-3	TE, NA	*bla*_CTX−M−27_	≥512	≥512	4	≥512	≥512	2	≥512	128	–	–	–	–	1, 2
C	4	AH-4-L-1	TE, NA, CIP	*bla*_CTX−M−14_	≥512	≥512	4	≥512	≥512	4	≥512	128	–	–	–	–	1, 2
C	4	AH-4-L-2	TE	*bla*_CTX−M−1_ + *bla*_TEM1_	≥512	≥512	8	≥512	≥512	2	≥512	256	–	–	–	–	1, 2
C	4	AH-5-L-3	TE, NACHL	*bla*_CTX−M−1_	≥512	≥512	8	≥512	≥512	2	≥512	128	–	–	–	–	1, 2
C	4	AH-11-L-3	NA, CIP, CHL	*bla*_CTX−M−27_ + *bla*_OXA−1_	≥512	≥512	8	≥512	≥512	8	≥512	128	–	–	+	–	1, 2
C	4	AH-10-L-2	TE, NA, CIP, CHL	*bla*_CTX−M−15_	≥512	≥512	8	≥512	32	32	≥512	128	+	–	–	–	1, 2
D	1	C3-L-4	NA, GM	*bla*_TEM1_	8	256	8	16	16	0.5	0.5	0.5	+	*dfr*A17+*aad*A5	+	–	1, 2
D	1	C9-L-2	TE, NA	*bla*_CTX−M−15_ + *bla*_TEM1_	≥512	≥512	8	≥512	≥512	4	≥512	256	–	–	+	–	1, 2
D	4	AC-4-L-1	TE, NA, CIP, CHL	*bla*_CTX−M−14_ + *bla*_CTX−M−15_ + *bla*_OXA−1_	≥512	≥512	8	≥512	≥512	16	≥512	256	–	–	+	–	1, 2
E	1	M4-L9	NA, CIP	*bla*_CTX−M−15_	≥512	≥512	16	≥512	≥512	≥512	≥512	256	–	–	+	–	1
E	1	M5-L4	TE, NA, CIP, GM, CHL	*bla*_TEM1_ + *bla*_CTX−M−14_	≥512	≥512	8	≥512	≥512	2	≥512	64	–	–	–	–	1, 2
E	1	M10-L-2	TE, NA, CIP, GM	*bla*_TEM1_	≥512	≥512	8	≥512	32	4	8	2	+	–	–	–	1, 2
E	1	M10-L-5	TE, NA, CIP	*bla*_CTX−M−1_	≥512	≥512	4	≥512	≥512	2	≥512	128	–	–	–	–	1, 2
E	4	AM-6-L-4	TE, SXT, NA, CIP	*bla*_CTX−M−15_	≥512	≥512	8	≥512	≥512	32	≥512	256	+	–	+	–	1, 2
E	4	AM-7-L-5	TE, SXT, NA, CIP, CHL	*bla*_CTX−M−14_	≥512	≥512	4	≥512	≥512	1	≥512	128	–	–	+	–	1, 2
E	4	AM-7-S-1	TE, NA, CIP, GM, CHL	*bla*_CTX−M−15_	≥512	≥512	8	≥512	≥512	16	≥512	64	–	–	–	–	2
E	4	AM-9-S-3	TE, NA, CIP, CHL	*bla*_SHV−12_	256	≥512	8	≥512	64	128	64	4	–	–	+	–	1, 2
E	4	AM-10-L-2	TE, NA, CIP, CHL	*bla*_CTX−M−15_	≥512	≥512	8	≥512	≥512	32	≥512	256	—	–	–	–	1, 2

Among the 49 3GC- or 4GC-resistant APEC isolates, 16 (32.7%) harbored class 1 integrons, and four (8.2%) contained different gene cassette arrangements, including *aad*A5+*dfr*A17 (*n* = 1), *aad*A1+*aad*A2+*dfr*A12 (*n* = 1), and *aad*A1+*aad*A2+*aad*A22+ *dfr*A12 (*n* = 2). However, 26 isolates (53.1%) harbored class 2 integrons, and only 1 isolate (2.0%) showed gene cassette arrangement *est*X+*sat*2+*sat*1.

Of the 49 isolates screened for four CRISPR loci, only one lacked all four loci. Moreover, both CRISPR 1 and 2 arrays were detected in most isolates (36 isolates; 73.5%), followed by CRISPR 2 (18.4%) and CRISPR 1 (4.1%). Interestingly, one isolate possessed four CRISPR loci simultaneously.

### Distribution of MDR and non-MDR in each CRISPR array

**Figure 1:**
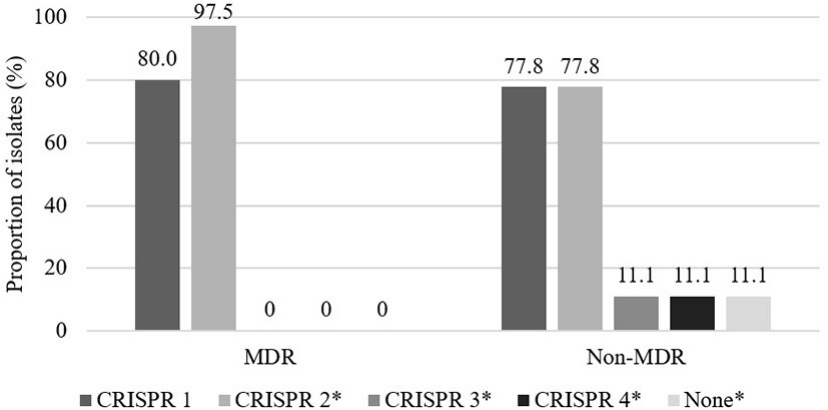
Distribution of CRISPR between multidrug resistant (MDR) and non-multidrug resistant (non-MDR) isolates in 49 third- or fourth-generation cephalosporin-resistant avian pathogenic *E. coli*. Multidrug resistance was defined as resistance to three or more antimicrobial classes. The asterisk indicates that prevalence between MDR and non-MDR isolates in each CRISPR array is significantly different (*p* < 0.05).

The distribution of MDR and non-MDR isolates with respect to CRISPR locus among the 49 3GC- or 4GC-resistant APEC isolates is presented in Figure 1. Although the distribution of CRISPR 1 showed no significant differences between MDR (80.0%) and non-MDR (77.8%) APEC isolates, CRISPR 2 was significantly more prevalent in MDR APEC isolates (97.5%) than in non-MDR APEC isolates (77.8%, *p* < 0.05). Moreover, CRISPR 3 and 4 were absent in MDR APEC isolates and were significantly more prevalent in non-MDR APEC isolates (each 11.1%, *p* < 0.05).

### Protospacer matching with plasmid and phage sequences

The protospacers matching the plasmids and phages sequences, are presented in Table 3. The most prevalent protospacer was associated with gene regulation, comprising 35.9% protospacers of CRISPR 1; followed by 13.4% of hypothetical proteins from the protospacers of CRISPR 1; 10.3 and 6.5% of phage proteins from the protospacers of CRISPR 1 and 2, respectively; and 6.5% of lysozyme inhibitor proteins from the protospacers of CRISPR 2. In CRISPR 3, two protospacers were associated with a protein encoding the terminase large subunit. None of the protospacers of the CRISPR arrays weas directly associated with antimicrobial resistance.

**Table 3:** Protospacer matching plasmids and phages, and spacer sequences in 49 third- or fourth- generation cephalosporin-resistant avian pathogenic *E. coli* isolated from five integrated broiler operations.

**CRISPR array**	**Name of Protospacer**	**Sequences (5′−3′)**	**No. of isolates (%)**	**Protospacer match**
CRISPR 1	6	TTTTGCTGACACCGGCAATACTGAACGGCTGG	13 (33.3)	DNA cytosine methyltransferase
	85	AAACAGATTGTTCGTTTTCCCCATATTCATGA	1 (2.6)	DUF1380 domain-containing protein
	88	CACATTCAACAGGTTAAGGTAACCGATTTGAC	3 (7.7)	Hypothetical protein
	91	GTTAAACCGTGATGATCAGCAAAAGCTATTTC	1 (2.6)	Phage capsid scaffolding protein
	94	GCTGGTGGCGCGGGCAAACGGAACAATCCCGC	1 (2.6)	*darB*, helicase
	97	CTGGATTTCACCTCAGCAAATGCTGGATGTGG	1 (2.6)	phage portal protein
	111	CGGAGGAAGTACCACGCCTGACCAGCGTTGAC	1 (2.6)	Hypothetical protein
	126	ACTCATACATCGATGACGGAGTAAGCAGTACG	2 (5.1)	Hypothetical protein
	128	TTCTGGGCGTTCCCTACCCCCGCCAGAAACGC	2 (5.1)	tail tape measure protein
CRISPR 2	20	TAATCCGATATCAGTGGGCGCCGCTTTACTGA	3 (6.5)	Lysozyme inhibitor *LprI*
	50	GCCGTCCAGAATTTTTTAAAGCGCTTCAACTG	1 (2.2)	putative capsid protein
	72	ATTAAATCGTCAGAAAATAGCGGTAATCAAGTC	2 (4.3)	tail collar fiber protein
CRISPR 3	5	AGGAAATTTCCGGCGACTAACGAAACAGGTTG	1 (100.0)	terminase large subunit
	6	GTCATCGGTATCGTCACGCGCAAGGGATTCAA	1 (100.0)	terminase large subunit

## Discussion

In general, the broiler industry is a vertically integrated production, processing, and distribution system in Korea. This allows individual producers to combine different approaches, such as biosecurity and sanitation practices, housing technologies, and feeding schedules, to enhance production rates and food safety (30, 31). Despite the mass broiler operations in Korea, APEC is a major cause of large economic losses, and intensive antimicrobial treatment is applied by operation systems (3). Moreover, previous studies have reported the relationship between vertically integrated production systems and increased antimicrobial use, resulting in antimicrobial resistance (32, 33). Thus, APEC isolates from chicken in Korea grown by vertically integrated production have shown increased level of resistance during 1985–2005 (34).

The systemic form of colibacillosis usually results in syndromes including respiratory signs or lameness and mortality (35). However, the causative bacterium (*E. coli*) is present in the farm environment, and it is a normal component of the poultry intestinal microbiota (36). Although previous studies have attempted to identify virulence markers to differentiate the pathogenic strains, infection by highly virulent APEC is also considered a secondary disease caused by opportunistic bacteria (37, 38). Therefore, colibacillosis frequently occurs in young chickens on most farms because of impaired host defenses (39). Moreover, although Kemmett et al. (40) have reported that the prevalence of virulence factors in APEC populations declines with age, in this study, the significant differences in the rates of colibacillosis in 4-week-old chickens among the integrated operations were potentially induced by host factors such as immunosuppression or prior infection, as well as management techniques including the farm environment.

In 2019, 3GCs and 4 GCs were declared as critically important classes of antimicrobials by WHO because of their importance to human health (9). Moreover, 3GC-resistant isolates are often multi-drug resistant and increase its potential threat to human and animal medicine (11, 31, 41). In this study, the prevalence of farms with 3GC- or 4GC- resistant APEC isolates varies from 15.8 to 53.3% in 1-week-old broilers, and 8.3–60.0% in 4-week-old broilers. Although 3GCs and 4GCs are not approved for use in poultry in Korea, high rates of resistance to 3GCs or 4GCs have been continuously reported (42). Odoi et al. (43) reported that the emergence of 3GC- and 4GC-resistant isolates is independent of the use of 3GCs and 4 GCs in farms. Further, UK (44) revealed that the use of other β-lactam compounds, such as AM, induces selective resistance against 3GCs and 4GCs as well as gene expression of ESBLs and/or pAmpC β-lactamases. In Korea, AM is the most common antimicrobial agent used to treat or prevent necrotic enteritis in 2–4-week-old broilers (42). Thus, the high proportion of 3GC- or 4GC- resistant APEC isolates in 1- and 4-week-old broilers may be attributable to the transmission of β-lactam resistant strains persistently maintained in hatchery and farm environments following the use of AM.

Since the introduction of 3GCs in medicine, gram-negative bacteria, especially Enterobacteriaceae such as *E. coli*, have developed resistance through the production of plasmid-mediated β-lactamase enzymes, such as ESBL and pAmpC β-lactamase. Therefore, the molecular characteristics of ESBL- and pAmpC β-lactamase-producing 3GC- or 4GC-resistant APEC isolates were analyzed in this study. All 49 3GC- or 4GC-resistant APEC isolates possessed one or more β-lactamase genes. In this study, the most prevalent ESBL-encoding genes were CTX-M group I and CTX-M group IV, which is similar to the previous findings in poultry and humans worldwide (31, 45–47). CTX-M types are often associated with coresistance phenotypes- particularly resistant to fluoroquinolones and aminoglycosides (48). Therefore, the existance of CTX-M groups in food animals could reduce the effectiveness of antimicrobial treatment against bacterial infection, as well as prevent the selection of appropriate therapeutic agents.

Interestingly, three isolates contained *bla*_SHV−12_. Although *bla*_SHV_ is one of the most prevalent genes conferring resistance to ESBL in *E. coli* (8, 49), *E. coli* containing *bla*_SHV_ in Korea has not been reported in poultry industries, such as layer hatcheries (11), chicken meat industry (31), and layer operations (41). Thus, emergence of *bla*_SHV−12_ in broiler farms may influence the dissemination of the gene among bacterial populations *via* plasmid-mediated horizontal gene transfer. Therefore, monitoring is essential to track the transmission of *bla*_SHV−12_ between different reservoirs in the poultry industry.

Gundran et al. (49) reported that the coexistence of different β-lactamase genes is associated with increased resistance. In this study, 14 (28.6%) isolates contained more than one β-lactamase gene, and the most common combination was *bla*_CTX−M−15_+*bla*_TEM−1_ (12.2%), followed by *bla*_CTX−M−14_+*bla*_TEM−1_ (4.1%). Bajpai et al. (50) reported that *bla*_TEM−1_ itself is not an ESBL or pAmpC β-lactamase; however, it can acquire ESBL activity through mutations that alter sequences of the amino acids adjacent to the active site of β-lactamase. In this study, isolates containing multiple β-lactamase genes exhibited higher MICs than those containing only *bla*_TEM1_. These results are in accordance with the findings of previous studies that the possession of ESBL and pAmpC genes increases resistance to cephalosprins and induces MDR (51, 52).

Class 1 and 2 integrons are highly associated with antimicrobial resistance gene cassettes in bacterial isolates, especially in the isolates of the Enterobacteriaceae family (53–55). In this study, 16 and 26 of the 49 β-lactamase-producing *E. coli* isolates harbored class 1 and 2 integron genes, respectively. In particular, four isolates with class 1 integron contained gene cassettes comprising *aadA* and *dfrA*, which are related with aminoglycoside and trimethoprim resistance, respectively (56, 57). Among the isolates harboring class 2 integron, only one contained a gene cassette comprising *estX* + *sat2* + *sat1*. Previous studies have reported that the presence of gene cassettes associated with antimicrobial resistance indicates increased resistance rates (58, 59) as well as increased potential for horizontal transfer (60).

*E. coli* comprises of four CRISPR loci, which are classified as Type I-E (CRISPR 1 and 2) or Type I-F (CRISPR 3 and 4), depending on the presence of the related gene *cas* (61). The relative prevalence of the CRISPR loci in this study was consistent with the findings of previous studies (62, 63). The prevalence of CRISPR 1 and 2 was high because these two loci have been preserved and stationary within *E. coli* over a long period, whereas the CRISPR 3 and 4 loci have recently emerged (26, 64). Interestingly, the prevalence of CRISPR 2 was significantly higher in MDR isolates, whereas CRISPR 3 and 4 were significantly more prevalent in non-MDR isolates. Further, Aydin et al. (65) and Kim and Lee (63) reported the overrepresentation of CRISPR I-F among susceptible isolates and the significantly higher prevalence of CRISPR 1 and 2 among MDR isolates. Although the CRISPR system is well known as an immune mechanism of prokaryotes (26, 66), CRISPR I-E system also plays several roles associated with functional abilities, such as gene regulation (67) and pathogenicity (68), by targeting bacterial chromosomes. Moreover, the CRISPR I-F system is considered to interfere with the acquisition of antimicrobial resistance plasmids, causing the isolate to be susceptible (65).

CRISPR arrays consist of diverse spacers, which are short sequences between each repeat, and even closely related isolates have different spacer compositions based on their previous encounters with different phages and plasmids (69). These spacers are derived from protospacers, which are short external sequences at specific locations that are inserted into the array during the process of infection. Thus, the protospacer identity can be crucial in revealing the roles of CRISPR (63). In this study, nine, three, and two protospacer matches were detected in CRISPR 1, 2, and 3, respectively, but none of the protospacers of CRISPR I-E and CRISPR I-F was directly associated with antimicrobial resistance. Our findings indicate that the distribution and characteristics of 3GC or 4GC-resistant APEC differed among the integrated broiler operations, and improved management protocols are required to control of the horizontal transmission of 3GC or 4GC-resistant APEC.

## Data availability statement

The original contributions presented in the study are included in the article/Supplementary material, further inquiries can be directed to the corresponding author.

## Ethics statement

Ethics approval regarding animal use was not necessary in this study because no live animal was used. The carcasses were collected from the farm and sent directly to the lab.

## Author contributions

HK, S-KL, and YL conceived of the presented idea. HK developed the theory and performed the experiments. HK and S-KL verified the analysis. HK wrote the manuscript with supervision from S-KL and YL. All authors contributed to the article and approved the submitted version.

## Funding

This work was supported by the Animal and Plant Quarantine Agency, Ministry of Agriculture, Food and Rural affairs, Republic of Korea (Grant Number Z-1543061-2021-23-02).

## Conflict of interest

The authors declare that the research was conducted in the absence of any commercial or financial relationships that could be construed as a potential conflict of interest.

## Publisher's note

All claims expressed in this article are solely those of the authors and do not necessarily represent those of their affiliated organizations, or those of the publisher, the editors and the reviewers. Any product that may be evaluated in this article, or claim that may be made by its manufacturer, is not guaranteed or endorsed by the publisher.
